# Totally Percutaneous Repair of an Aortic Arch Dissection: A Case Report

**DOI:** 10.21470/1678-9741-2022-0094

**Published:** 2023

**Authors:** Eduardo Keller Saadi, Ana Paula Tagliari, Rodrigo Petersen Saadi

**Affiliations:** 1 Postgraduate Program in Cardiology and Cardiovascular Sciences, Universidade Federal do Rio Grande do Sul, Porto Alegre, Rio Grande do Sul, Brazil.; 2 Cardiac Surgery Department, Hospital São Lucas da Pontifícia Universidade Católica do Rio Grande do Sul, Porto Alegre, Rio Grande do Sul, Brazil.; 3 Cardiac Surgery Department, Hospital de Clínicas de Porto Alegre, Porto Alegre, Rio Grande do Sul, Brazil.; 4 Cardiac Surgery Department, Hospital Mãe de Deus, Porto Alegre, Rio Grande do Sul, Brazil.

**Keywords:** Aneurysm, Aortic Thoracic, Prostheses and Implants, Case Report

## Abstract

Although the endovascular repair of descending thoracic aorta diseases is an already consolidated procedure, this approach is not well-established for ascending aorta and arch pathologies. A 71-year-old male patient who had undergone an open ascending aorta replacement ten years ago presented with a huge dissected aortic arch aneurysm. Vascular accesses were obtained with ultrasound-guided punctures, followed by aortic arch exclusion using aortic endoprostheses and the chimney-graft technique for preserving supra-aortic branches flow. This case demonstrates the feasibility of a totally percutaneous aortic arch repair provided that careful preprocedural planning and a dedicated team are available for such a challenging intervention.

## INTRODUCTION

Even though the endovascular approach for descending thoracic aorta pathologies has been consolidated as an effective and safe procedure, this is not the case for ascending aorta and aortic arch diseases, which are considered high-complexity and high-morbimortality procedures.

However, in a group of high-selected patients, with feasible anatomy, the aortic arch endovascular approach has become an alternative to conventional open cardiac surgery. Herein, we present a case report of a patient with a dissected aortic arch aneurysm treated by a totally percutaneous endovascular approach using the chimney technique and aortic thoracic endoprostheses.

## CASE PRESENTATION (MOVIE 1)

**Fig. 1:**
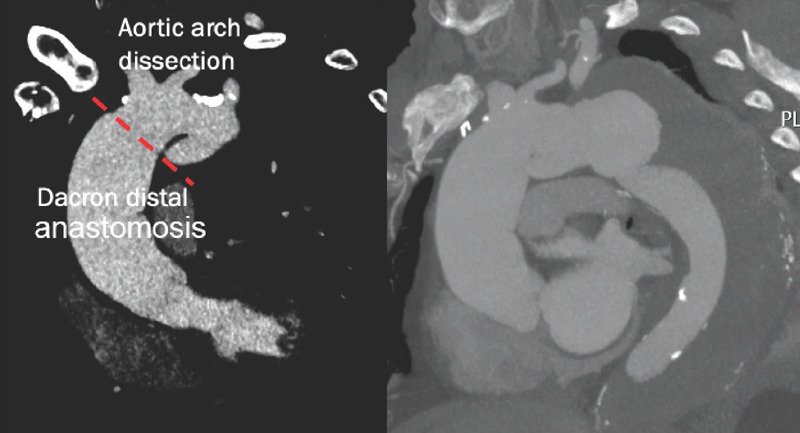
Preoperative computed tomography angiography showing a huge aortic arch aneurism and dissection.

A 71-year-old male patient presenting acute Stanford type A aortic dissection had undergone an open ascending aorta replacement under cardiopulmonary bypass through right axillary artery cannulation 10 years ago. In the late postoperative course, he developed a sternal surgical wound infection, and some steel wires had to be removed. At the begging of 2022, he presented a sudden severe upper back pain, alleviated 24 hours after painkillers administration and arterial pressure and heart rate control. At this point, he was transferred to our tertiary hospital with a computed tomography angiography showing a huge aortic arch aneurysm (76 mm) and an aortic tear starting close to the distal anastomoses of the ascending aorta with the Dacron® graft, in the inner aspect of the aorta curvature (Figure 1). All the main arterial branches but the left renal artery originated from the true lumen. After institutional heart team discussion, taking into consideration the high risk of a redo open cardiac surgery, an endovascular approach was chosen.

**Video 1:**
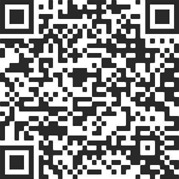
Totally percutaneous aortic arch dissection repair using thoracic endovascular grafts and VIABAHN® stents.

**Fig. 2:**
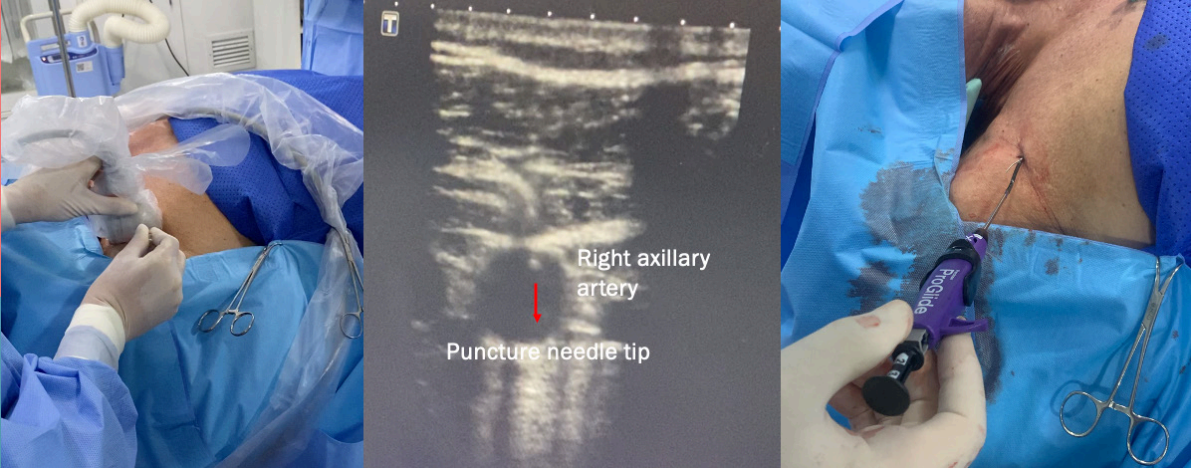
Ultrasound-guided right axillary artery puncture and Perclose/ProGlide™ insertion.

**Fig. 3:**
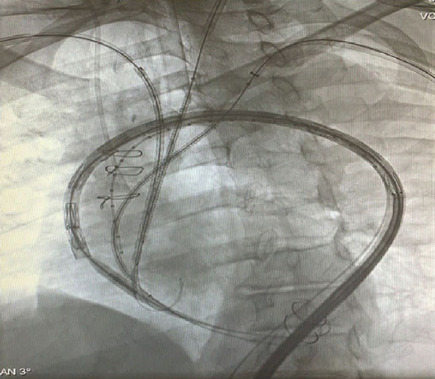
VIABAHN® endoprosthesis and thoracic stent graft positioned into the brachiocephalic trunk, left carotid artery, left subclavian artery, and aortic arch.

**Fig. 4:**
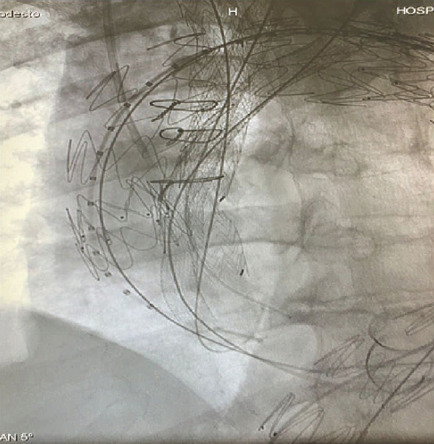
Final result showing all the endoprostheses deployed.

Under general anesthesia, both axillary arteries and the left common carotid artery were totally percutaneously accessed with ultrasound-guided punctures, and Perclose/ProGlide™ suture-mediated closure devices (Abbott Vascular Devices, Redwood City, California, United States of America) were employed (Figure 2). After systemic heparinization (100 IU/Kg, activated clotting time > 300 seconds), a 12F sheath was placed into the right and left axillary arteries, and an 11F sheath into the left common carotid artery. Hydrophilic stif guidewires (0.035 mm) were placed through the sheaths into the ascending aorta and left *in situ.* Then, VIABAHN® stent (GORE® VIABAHN® endoprosthesis; Gore, Flagstaf, Arizona, United States of America), with 100 and 150 mm in length, were positioned from the left common carotid and left subclavian arteries until the tip of the stents reached the ascending aorta. A 16×135 mm branched stent graft (GORE® EXCLUDER® Contralateral leg endoprosthesis) was inserted through the right axillary artery, positioned from the brachiocephalic trunk into the ascending aorta. An angiographic pigtail catheter was inserted through a 6F right femoral sheath, and a control angiography was performed. Then, a 46-42×233 mm thoracic endovascular graft (Cook Zenith Alpha™; Cook Inc., Bloomington, Indiana, United States of America) was advanced through a Lunderquist® guidewire (Cook) placed in the left ventricle from the left femoral artery access (Figure 3). All the grafts and stents were delivered at the same time. Finally, a second 44×233 mm thoracic endovascular graft (Cook Zenith Alpha™) was positioned and deployed distally to the previous one, to extend the distal thoracic aorta covered zone. Two additional stents, with 100 and 80 mm in length, were deployed in the left subclavian and in the left common carotid artery, respectively. The reason to use a second stent was the unavailability of longer VIABAHN® in an urgent context, demanding two stents to achieve the final required length. Final control angiography confirmed the complete exclusion of the aortic arch dissection and proper flow through all the supra-aortic branches (Figure 4). The patient was extubated in the operating room, had no neurologic complications, and was discharged 10 days after the procedure. Computed tomography angiography performed one week later showed complete exclusion of the aortic arch dissection.

## DISCUSSION

Conventional open surgery has been the gold-standard treatment for aortic arch aneurysms, but it requires cardiopulmonary bypass and hypothermic circulatory arrest, and it is associated with substantial morbidity and mortality. Consequently, over 40% of patients are considered unft for conventional open surgery^[^1^]^.

In this context, endovascular repair of aortic arch aneurysms has arisen as an attractive and less invasive option, especially for high-risk and with suitable anatomy patients, with fenestrated grafts, *in situ* fenestration, or branched grafts being the most commonly available options to be used in these cases^[^2^]^.

However, most of these options are custom-made devices, requiring a long manufacturing time and having a high cost. Furthermore, *in situ* fenestration needs a temporary bypass to perfuse the supra-aortic vessels during the procedure; fenestrations made in the graft fabric may result in fabric tearing and deformation of the stent-graft strut; and branched stent grafts are not readily available, limiting their use in urgent scenarios^[^3^, ^4^, ^5^]^.

Therefore, a less time-consuming alternative, particularly in acute scenarios, such as the presented here, would be to deploy a thoracic endovascular graft combined with chimney-stents deployment in parallel with the main aortic endograft, which allows an easy of-the-shelf solution to preserve the supra-aortic branches flow and to permit proximal extension of the landing zones^[^2^]^.

By definition, the chimney technique involves the placement of bare-metal stents or covered stents in parallel to the main aortic stent graft^[^6^,^7^]^, and one of its main advantages is to be the only total endovascular approach available in emergency situations^[^8^]^. Therefore, we decided to use the parallel graft technique to maintain antegrade flow into the supra-aortic vessels.

Another interesting point of this case is that we used a totally percutaneous approach by performing ultrasound-guided axillary and carotid artery punctures, combined with suture-mediated closure devices employment (Perclose/ProGlide™), a technique that, although considered “of label”, contributes to reduce procedural time and morbidity.

## CONCLUSION

This case report contributes to demonstrate that a totally percutaneous approach for aortic arch pathologies is feasible in selected patients at high risk for conventional surgery. Therefore, the use of chimneys and aortic endoprostheses, provided that carefully planned and carried out by a dedicated experienced team, can be an alternative to manage one of the most challenging aortic pathologies without any surgical incision.
